# Application of receptor models on water quality data in source apportionment in Kuantan River Basin

**DOI:** 10.1186/1735-2746-9-18

**Published:** 2012-12-10

**Authors:** Mohd Fahmi Mohd Nasir, Munirah Abdul Zali, Hafizan Juahir, Hashimah Hussain, Sharifuddin M Zain, Norlafifah Ramli

**Affiliations:** 1 Department of Environmental Sciences, Faculty of Environmental Studies, UPM Serdang, Selangor, Malaysia; 2 Department of Environment, Federal Government Administrative Centre, Environment Institute of Malaysia, Putrajaya, Malaysia; 3 Department of Chemistry, Faculty of Science, Universiti Malaya, Kuala Lumpur, Malaysia; 4 Surface Water Monitoring Unit, Water and Marine Division, Department of Environment Malaysia, Federal Government Administrative Centre, Putrajaya, Malaysia

**Keywords:** Water quality, Receptor modeling, Multiple linear regression (MLR), Artificial neural network (ANN)

## Abstract

Recent techniques in the management of surface river water have been expanding the demand on the method that can provide more representative of multivariate data set. A proper technique of the architecture of artificial neural network (ANN) model and multiple linear regression (MLR) provides an advance tool for surface water modeling and forecasting. The development of receptor model was applied in order to determine the major sources of pollutants at Kuantan River Basin, Malaysia. Thirteen water quality parameters were used in principal component analysis (PCA) and new variables of fertilizer waste, surface runoff, anthropogenic input, chemical and mineral changes and erosion are successfully developed for modeling purposes. Two models were compared in terms of efficiency and goodness-of-fit for water quality index (WQI) prediction. The results show that APCS-ANN model gives better performance with high *R*^*2*^ value (0.9680) and small root mean square error (RMSE) value (2.6409) compared to APCS-MLR model. Meanwhile from the sensitivity analysis, fertilizer waste acts as the dominant pollutant contributor (59.82%) to the basin studied followed by anthropogenic input (22.48%), surface runoff (13.42%), erosion (2.33%) and lastly chemical and mineral changes (1.95%). Thus, this study concluded that receptor modeling of APCS-ANN can be used to solve various constraints in environmental problem that exist between water distribution variables toward appropriate water quality management.

## Introduction

**Figure 1:**
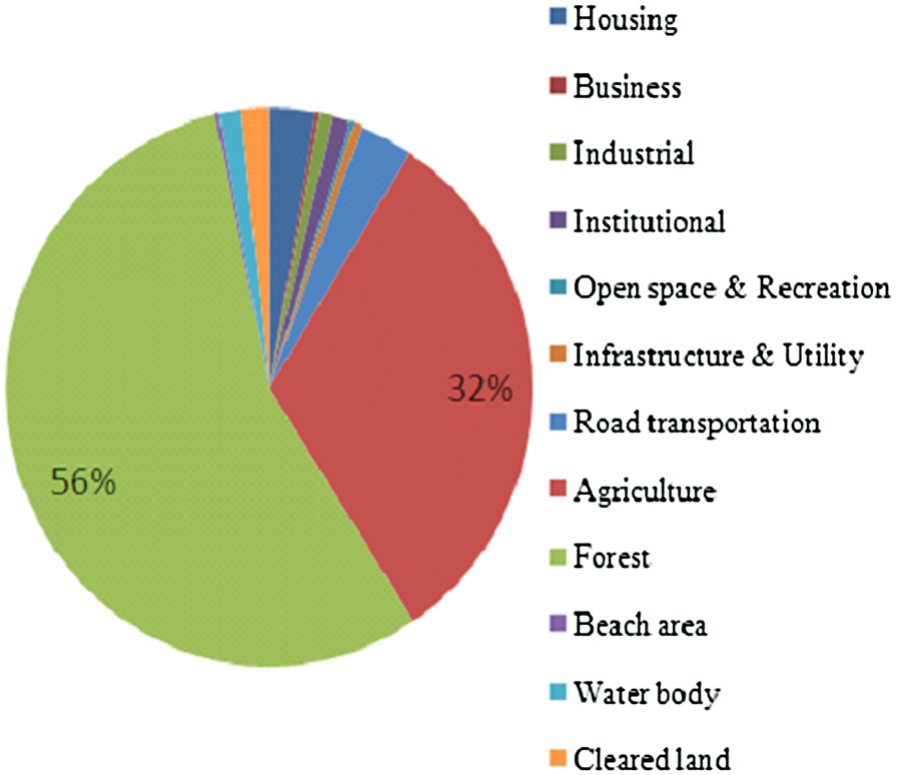
Percentage of land use in Kuantan River Basin.

Surface water quality studies are among the preliminary topics in Malaysia which provide an overview on the status of the specified river. In addition, surface waters are most susceptible due to easy accessibility for wastewater (Singh *et al.,*[1]) and anthropogenic activities from its vicinity. Although 60% of the main rivers in Malaysia are regulated for domestic, agricultural and industrial fields (DID, [2]); sewage disposal, industrial effluents (Rosnani, [3]) and urbanization are among the major pollution sources influencing the health of the rivers in Malaysia (Figure 1). Monitoring and the study of surface water will then offer judgments on the authorities to the offender and the concerns of researchers in the field of ecotoxicology and risk assessment if other contaminants such as inorganic and organic micropollutants that may affect water quality.

Monitoring programs often worked out with frequent water samplings at many sampling sites all over the world and determination of physiochemical parameters can provide a representative and dependable estimation of the surface water quality. In Malaysia, Department of Environment (DOE) has been conducting unstoppable monitoring activities since 1978 resulting to large data matrix collection and desperately requires remarkable statistical tools such as multivariate and artificial intelligent for exceptional data illustration.

The program covered initially all the river basin in Malaysia, involving mainly manual sampling and *in-situ* measurements of the river water quality. According to the DOE’s Environmental Quality Report in 2007, 158 river basins are involved in this program to monitor river quality changes on a continuous basis (DOE, [4]). Even though DOE have a regular monitoring program to provide the complex environmental data sets, however they are is still lacking in the application of multivariate statistical methods. This is in attempt to extract all possible information from the river water quality data sets and consequently determine the major sources that influencing the river class at Kuantan River Basin. The multivariate statistical technique and exploratory data analysis are the appropriate tools for a meaningful data reduction and interpretation of multi-constituent chemical and physical measurement (Massart *et al.*, [5]).

Water quality is referring to the characteristics of water whether in its physical, chemical or biological character. Based on the water quality data, the water quality index (WQI) was developed to evaluate the water quality status and river classification in Malaysia. WQI provides a useful way to predict changes and trends in the water quality by considering multiple parameters. WQI is formed by six selected water quality variables, namely dissolved oxygen (DO), BOD, chemical oxygen demand (COD), SS, AN and pH (DOE, [6]). WQI values are in the range 0–100. If the values are in the range of 81–100 the samples water analyzed in the specific station fall in clean category. Values ranging from 60–80 and 0–59 are grouped as slightly polluted and polluted area respectively.

Continuous monitoring of river water quality reveals the chemical and physico-chemical parameters for the interpretation of large data set with many variables; therefore environmetric approach need to be constructed to comprehend the variation on the data since it is not entirely convincing. In this study, the large data matrix obtained from monitoring programme conducted by DOE, Malaysia from year 2003 to 2007 was introduced to receptor models techniques that involved varimax factor from principal components analysis (PCA) with two different data based on multiple linear regression (MLR) and artificial neural network (ANN) models. These approaches were conducted in many fields such as prediction of ozone concentrations (Bandyopadhyay and Chattopadhyay, [7]; Sousa *et al.*, [8]), forecasting summertime (Chaloulakou *et al.*, [9]), prediction medical waste generation (Jahandideh *et al.*, [10]), prediction the lower heating value of municipal solid waste (Ogwueleka and Ogwueleka, [11]) however emphasis in water quality were not yet steady especially in tropical regions. The development of such mathematical tools will facilitate an early warning for people whom reside near the river other than to environmental agencies in order to protect and conserve the river from further being soiled by pollutions.

Source apportionment techniques were applied in the data set by combining PCA with MLR and PCA with ANN. The aim of this study is to discover the major pollution sources that significantly change the WQI values in Kuantan River Basin from the varimax factors produced for MLR and ANN models. The uncorrelated new variables that account much of the original data will be used as input variables for the models; other than combining statistical and an artificial intelligent techniques which has been received great spotlight in environmental pattern recognition study (Sousa *et al.*, [8]). Moreover the particular discussions on comparison for both techniques were not extensively reported in water quality study. Thus, this study will determine the models that best fit on the entire data sets by performing non-linear transformation of input data (resulted VFs) to approximate WQI values.

Generally, MLR may lead to incorrect identification of most the predictor due to collinearity between the input variables (Thompson *et al.*, [12]). Many study emphasizes the comparison between model result in good prediction performance of ANN, whilst the used of linear models are of the less efficient model that might attribute to the non-linearity of the data sets (Thompson *et al.*, [12]; Chaloulakou *et al.*, [9]; Bandyopadhyay and Chattopadhyay, [7]; Sousa *et al.*, [8]; Jahandideh *et al.*, [10]; Gutierrez-Estrada and Bilton, [13]; Rossel and Behrens, [14]; Wu *et al.*, [15]). ANNs are greatly suited to dynamic non linear system modelling (Mirsepassi, [16]) and have advantages over conventional simulation methods have been discussed in detail by French *et al.*, [17]. MLR also allows the reduction of the dimensionality of non-linear data set by correction amongst a large number of variable in terms of underlying factors without neglecting any information from the original data set (Juahir *et al.*, [18]). Although linear regression was one of the oldest statistical modelling techniques, their applications were still widely used in many linear relationships works. However its application in water quality study were less published and lead to this study aimed to certify whether this model were applicable for WQI forecasting in Kuantan River Basin. Despite the fact that many studies performed concluded that there is no general best modelling techniques, it still depends on the scope and objectives of the studies (Aertsen *et al.*, [19]).

The objectives of this study are to predict WQI values as well as to estimate the main contributor using MLR and ANN model from the varimax factors generated by PCA. This study will provide comprehensive understanding on goodness and weakness for both models and consequently finalised the correct model for WQI prediction in the Kuantan River Basin.

## Materials and methods

### Study area

**Figure 2:**
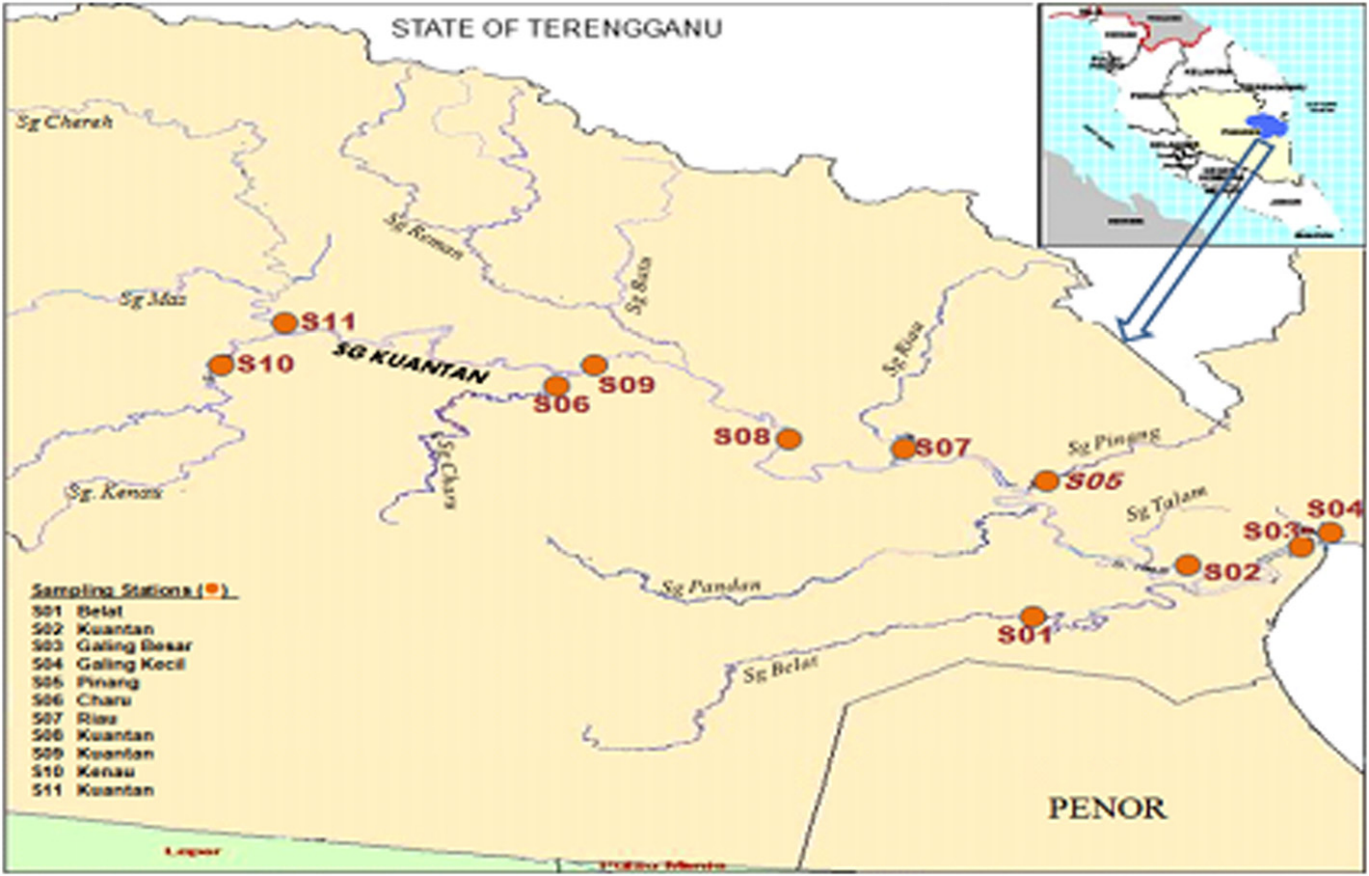
Monitoring station at Kuantan River Basin.

Kuantan River Basin is in the district of Kuantan at the north eastern end of Pahang State in Peninsular Malaysia (Figure 2). It is one of the important river basins in Pahang and covers an area of 1630 km^2^ catchment area which started from forest reserved area in Mukim Ulu Kuantan through agricultural areas, Kuantan town (state capital of Pahang) towards the South China Sea. Kuantan River Basin consists of several important tributaries and these rivers drain the major rural, agricultural, urban and industrial areas of Kuantan District and discharge into South China Sea.

Kuantan River basin which is in Kuantan District area has six administrative mukims (small district). In terms of land use, the main types of land use in this district are forest and agriculture that cover approximately 56% and 32% respectively, from the whole area of Kuantan District. Majority of the forested areas are at the west of Kuantan District or in upstream of the basin. Besides that, there is an ex-tin mining land in Sungai Lembing or at upstream or low sub basin area. The mining activities was started in 1906 and stopped in 1986 due to economic recession in our country.

**Figure 3:**
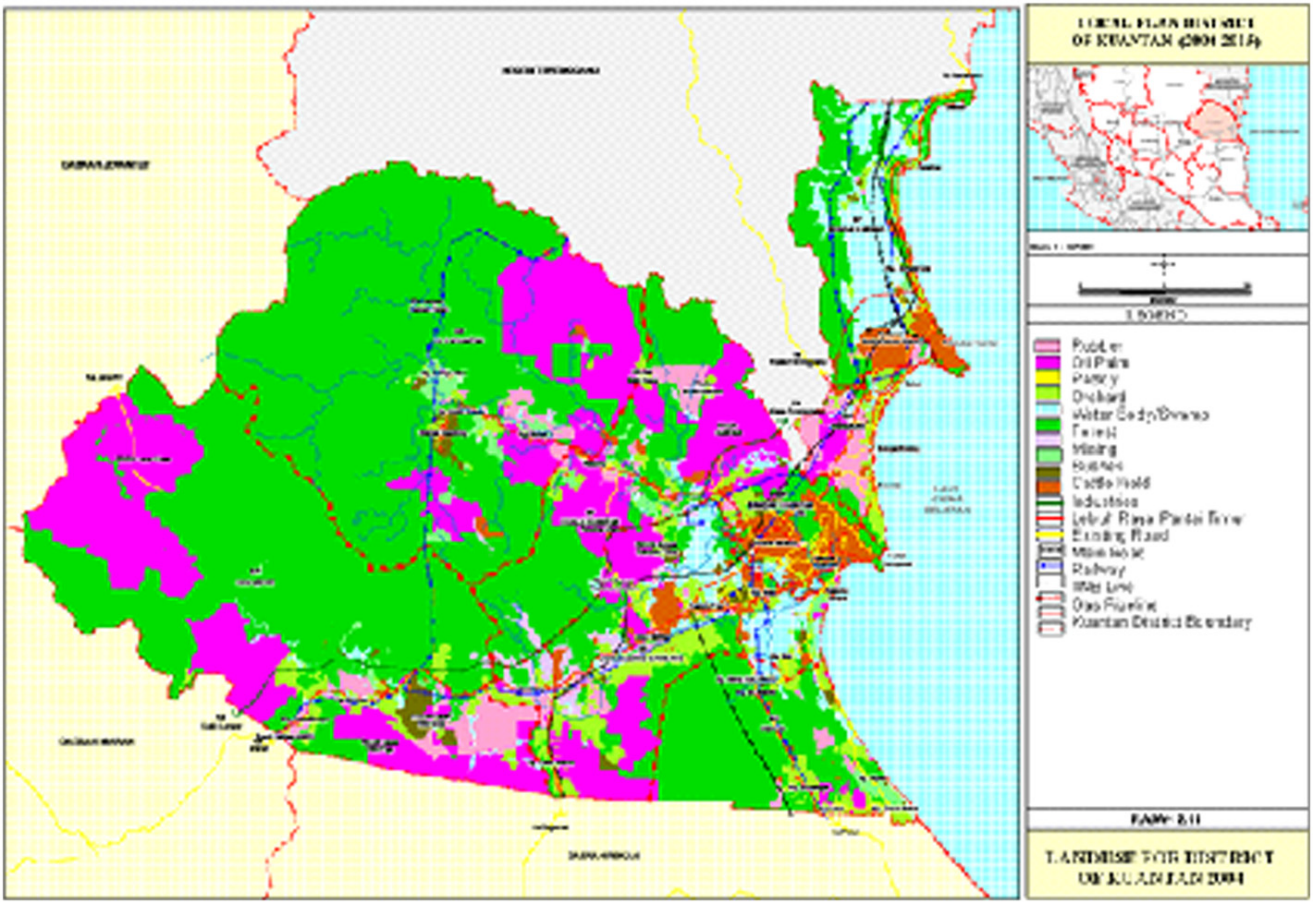
Land-use of Kuantan River Basin.

In term of land use utilisation, agriculture is considered to be one of the main economic activities in this river basin where it covers about 70,128 hectares of the area (DOE, [20]). According to land use map of Kuantan District as shown in Figure 3, main agricultural area is located in the middle part of the basin. The oil palm is the main agricultural crops (57,863 hectares), followed by rubber (10,191 hectares). Fruits are the third largest agricultural crops which covers the total area of 1,489 hectares.

There are three palm oil mills which are the major agro-based industries in the middle of the basin and might be contributed to deterioration of Kuantan river water quality (DOE, [21]). Recently, 42 tributaries in Peninsular Malaysia have been categorized as very polluted (Aiken *et al.*, [22]). Since 1999, there were 13 polluted tributaries all over Malaysia with 36 polluted rivers due to human activities such as industry, construction and agriculture (DOE, [20]).

### Data and parameters

The water quality data were collected in 2003 to 2007 from eleven monitoring stations provided by DOE. However, some of the stations are inconsistently sampling thus leading to the missing data. Thirty water quality parameters are selected by DOE in order to represent the water quality in the river. Unfortunately only 13 parameters are consistently sampled along 2003 to 2007 therefore a total of 275 observations were used for source apportionment and modeling techniques. The thirteen water quality parameters are selected for analysis in this study are: pH, dissolve oxygen (DO), biological oxygen demand (BOD), chemical oxygen demand (COD), suspended solid (SS), ammoniacal nitrogen (AN), dissolved solids (DS), total solids (TS), nitrate (NO_3_), chloride (Cl^-^), phosphate (PO_4_^-^), *Escherichia coli* (*E. coli*) and coliform. According to DOE [6], the water quality index (WQI) was developed to evaluate the water quality status and river classification. WQI consists of six selected water quality parameters known as DO, BOD, COD, SS, AN, and pH which provides useful way to predict the changes and trends in the water quality (DOE, [6]).

### Data preprocessing

The data were initially arranged according to the stations and year of monitoring. Variables that are not have been detected (below detection limit) were set to half of its detection limit in order to ensure that there is no missing data in the dataset. Normality test were performed using the Anderson-Darling test since the multivariate statistical techniques requires the variables to be normally distributed (Zhou *et al.*, [23]). Data that are not normally distributed undergo pretreatment which consist of centering, standardization and log-scaling method. Standardization opts to increase the influence of variables with small variance and vice versa (Krishna *et al.*, [24]). Log scaling was used upon variables which exhibit too low or high values (Felipe-Sotelo *et al.*, [25]). Statistical computation of PCA and MLR were carried out using XLSTAT 2010 Excel add-in Window software and prediction model of ANN was conducted by using JMP8 for Windows software (Camdevyren *et al.*, [26]).

### Principal component analysis (PCA)

The most powerful technique for pattern recognition that attempts to explain the variance of a large set of inter-correlated variables and transforming it into smaller set of independent (uncorrelated) variables (principal components). PCA aims to uncover a more underlying set of factors that accounts for the major pattern across all the original variables (Saim *et al.*, [27]). Moreover PC also, present information on the most meaningful parameters, which define whole data, set affording data reduction with minimum loss of original information (Krishna *et al.*, [24]). This technique provides information on the most significant parameters by rendering data reduction with minimum loss of original information (Vega *et al.*, [28]; Helena *et al.*, [29]; Wunderlin *et al.*, [30]). PCA is sensitive to outliers, missing data, and poor linear correlation between variables due to inadequate assigned variables (Sarbu and Pop, [31]). Hence, pretreatment data is required for a clearer image in the complex dataset. The principal component (PC) is expressed as

(1) $$y_{\mathrm{ab}}=z_{a1}x_{1b}+z_{a2}x_{2b}+z_{a3}x_{3b}+\ldots +z_{\mathrm{ai}}x_{\mathrm{ib}}$$

Where *z* is the component loading, *y* is the component score, *x* is the measured value of a variable, *a* is the component number, *b* is the sample number, and *m* is the total number of variables. PCA was performed on correlation matrix of rearranged data which explains the structure of the underlying dataset. The correlation coefficient matrix measures the variance of each constituent explained by relationship with each others. PCA of the normalized variables were then performed to extract the significant PCs and reduce the variables with minor significance. These PCs were subjected to varimax rotation (raw) generating VFs as it sometimes not readily interpreted thus performing varimax rotation is recommended to reduce the dimensionality of the data and identify most significant new variables. Varimax factor (VF) coefficient having a correlation >0.75 are regarded as strong significant factor loading (Liu *et al.*, [32]). Meanwhile VF in the range of 0.75-0.50 and 0.50-0.30 are considered as moderate and weak factor loading, respectively.

### Absolute principal component scores-multiple linear regression (APCS-MLR)

Receptor modeling application based on APCS-MLR is a commonly apply statistical technique for source apportionment of environmental contaminants in air pollution studies (Swietlicki and Krejei, [33]; Fung and Wong, [34]; Simeonov *et al.*, [35]; Simeonov *et al.*, [36]). It has been newly employed to water pollution source apportionment worldwide. It is based on the assumption that the total concentration of each contaminant is made up of the linear sum of elemental contributions from each of the pollution source components collected at the receptor site:

(2) $$Z_{\mathrm{bc}}=\sum Q_{\mathrm{ab}}R_{\mathrm{bc}}$$

Where *Z*_*bc*_ is the normalized concentration of contaminant (variable), *Q*_*ab*_ refers to the factor loadings, the coefficient matrix of the components relates with pollution sources and their elemental concentrations; and *R*_*bc*_ the factors cores in Eq. (2). *Q*_*ab*_ is dimensionless. Since, *Z*_*bc*_ in Eq. (2) is normalized value of variables, it cannot be used directly for computation of quantitative source contributions, the normalized factor scores determined in Eq. (2) were converted to unnormalized APCS following the method reported elsewhere (Thurston and Spengler, [37]). The contribution from each factor was then estimated by MLR, using the APCS values as the independent variables and the measured concentration of the particular contaminant as the dependent variable, as:

(3) $$M_{\mathrm{bc}}=d_{a0}+\sum D_{\mathrm{ab}}{\left(\mathrm{APCS}\right)}_{\mathrm{bc}}$$

Where *M*_*bc*_ is the contaminant’s concentration; *d*_*a0*_ is the average contribution of the *b*^th^ contaminant from sources not determined by PCA/FA, *D*_*ab*_ is the linear regression coefficient for the *a*^th^ contaminant and the *b*^*th*^ factor, and (APCS)_*bc*_ the absolute factor score for the *b*^th^ factor with the *c*^th^ measurement. The values for *M*_*bc*_, *d*_*a0*_ and *D*_*ab*_ have the dimensions of the original concentration measurements. After determining the number and identity of possible sources influencing the river water quality by PCA/FA, source contributions were computed through APCS-MLR technique. Quantitative contributions from each source for individual parameter or contaminant were compared with their measured values.

### Absolute principal component scores-artificial neural network (APCS-ANN)

Several studies on water quality model have been developed in order to manage and protect the water quality in many countries. Most of the models demand many inputs for model development and eventually lead to time consuming and high priced. ANN models are defined by topology, node characteristics and training or learning rules. It is an inter-related set of weights that composed of the knowledge generated by the model. An ANN contains large number of simple processing units, each interconnecting with others via excitatory or inhibitory connections. The most unique features of ANN model is the non-linear modeling capability, ability dealing with large sets amount of data and robustness to noisy data (Moatar *et al.*, [38]). The distributed representation over large number of unit together with interconnectedness among processing units, provide a fault tolerance. Three difference layers can be distinguished:

(i) An input layer which is connecting the input information to the network. In this assessment thirteen input nodes representing the thirteen water quality parameters were applied (DO, BOD, COD, SS, pH, AN, DS, TS, NO_3_, Cl^-^, PO_4_, *E. coli* and coliform).

(ii) Hidden layer which is acting as the intermediate computational layer. Multi-layer feed forward network formed by only one hidden layer. ANN models consist of the following set of equations:

(4) $$\begin{array}{cc} M_{b}=f\left[\sum P_{\mathrm{ab}}R_{a}\right] & 1\leq b\leq B-1 \end{array}$$

*P* represents the scaled input vector and *M* is output vector of the neurons contain in the hidden layer. The bias is set equal to 1.

(iii) Output layer is producing the desired output which is in this case the WQI following this equation:

(5) $$\begin{array}{cc} X_{c}=f\left[\sum P_{\mathrm{bc}}M_{b}\right] & 1\leq k\leq K \end{array}$$

The coefficients *P*_*ab*_ and *P*_*bc*_ in the summation, which are usually referred as the weights, are the fitting coefficients of the neural model.

**Figure 4:**
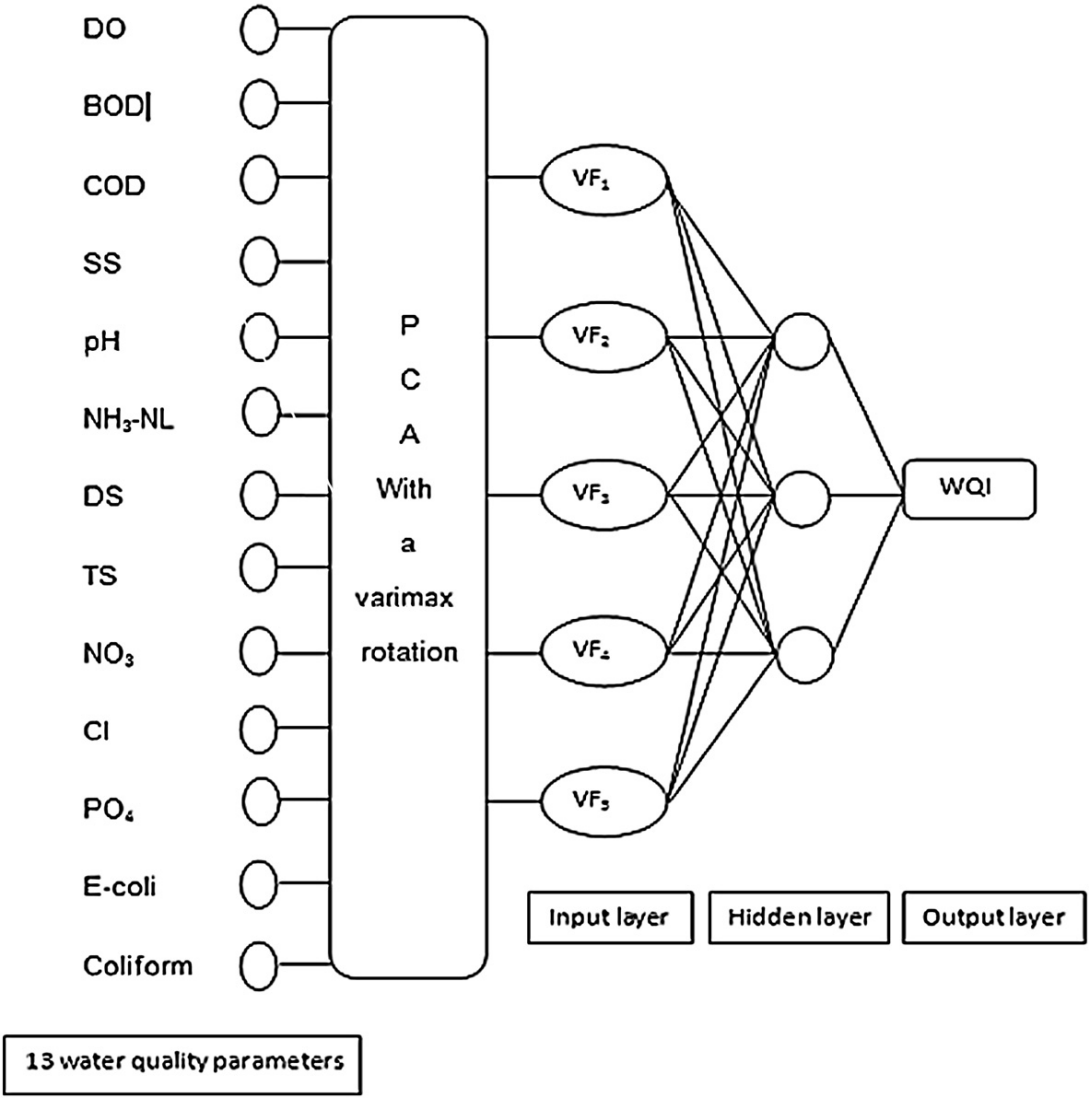
Example of ANN structure.

Many studies already emphasized the use of linear regression in apportionment of sources toward the dependable variables in their studies. Nonetheless, ANN application was yet to be explored. The aim of this method is to minimize the effect of multicollinearity and achieve better prediction model with minimum residual errors. The small number of input variables from APCS was combined with the ANN model to obtain high interpretability as the irrelevant and superfluous variables were excluded (Figure 4).

## Results

### Principle component scores (PCS)

The PCA showed that the two main PCs accounted for 45.13% of the total variance (PC1 21.40%; PC2 23.73%) for the overall observations. The larger variability graph for factor loading 1 and factor loading 2 were plotted to explain the variance. The 13 variables were well represented on the plane.

**Figure 5:**
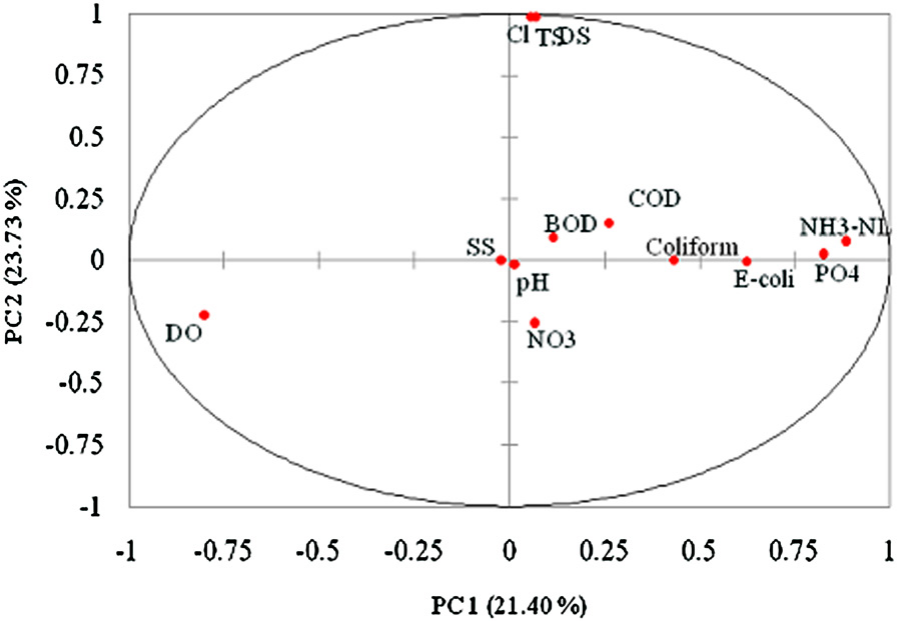
Variables (PC1 and PC2: 45.13%) after varimax rotation.

**Figure 6:**
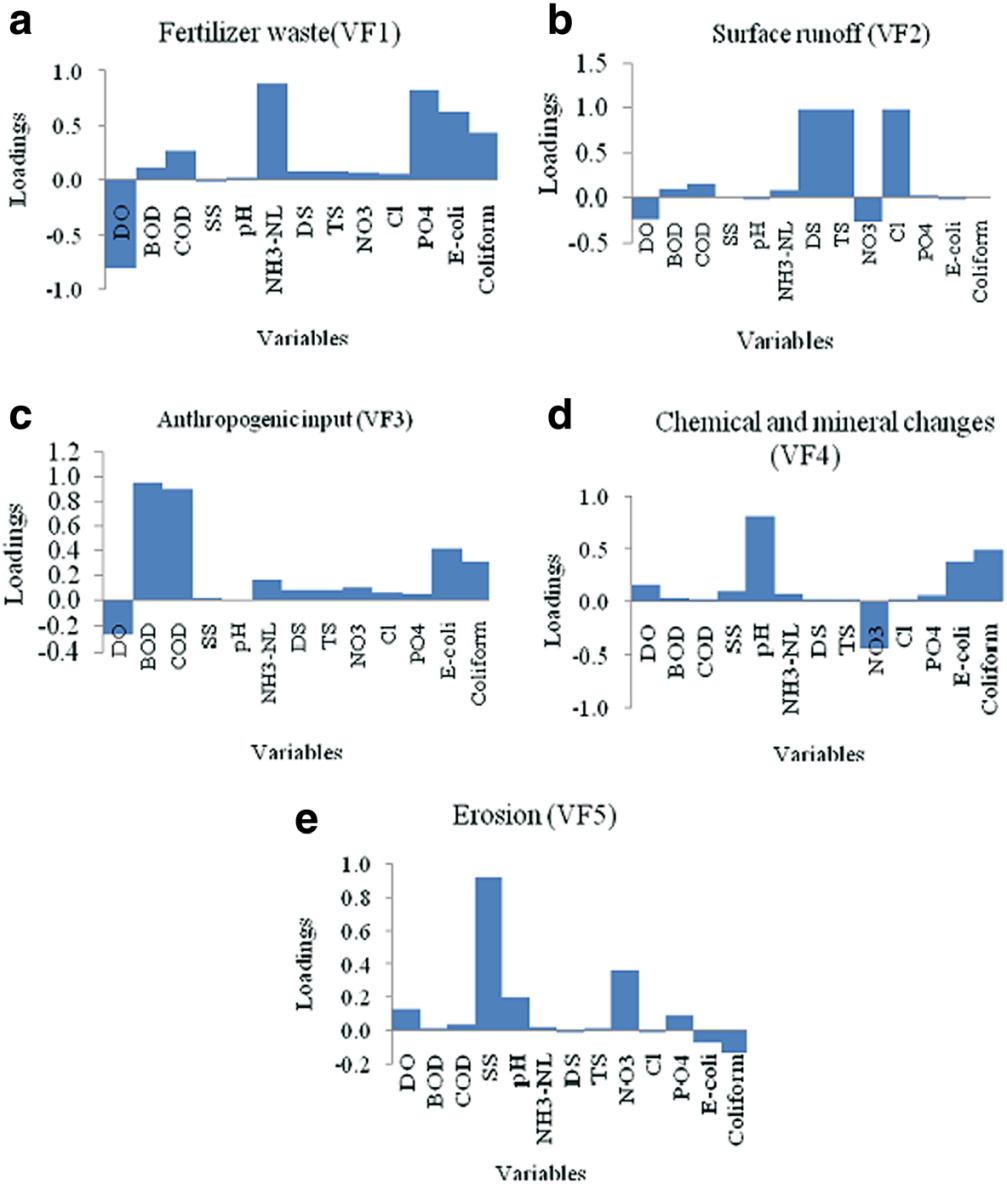
Graph plotting after varimax rotation: (a) Fertilizer waste (VF1); (b) Surface runoff (VF2); (c) Anthropogenic input (VF3); (d) Chemical and mineral changes (VF4); (e) Erosion (VF5).

The five VFs were generated after varimax rotation based on eigenvalues >1 (Kim and Mueller, [39]), (Figure 5). Eigenvalues and the corresponding factors were sorted by descending order and the initial variability was represented in percentage. The main approach of PCA is to reduce the number of variables by identifying the structure which corresponded between variables and classify the new variables as shown in Figure 6. PCA is competent to extract latent information and explains the structure of the data in detail (Wu *et al.*, [40]). PCA after varimax rotation indicates five VFs with 79.41% of the total variability (Table 1).

**Table 1:** The variability of VFs

**VF**	**D1**	**D2**	**D3**	**D4**	**D5**
Eigenvalue	4.213	2.656	1.249	1.184	1.022
Variability (%)	32.409	20.43	9.605	9.11	7.858
Cumulative %	32.409	52.839	62.444	71.554	79.412

### Source apportioning by absolute principal component scores (APCS)

PCA aims to exclude redundant information from the original raw dataset by obtaining a small number of variables. This is comprehensible especially for detailed analysis such as modeling. Source apportioning are well known especially for air pollution and water quality data as it integrated with WQI although it is less documented in tropical regions. In air pollution studies, PCA and environmetric techniques are used extensively to determine possible natural and anthropogenic contributions in the determination of total mass and concentration (Randolph *et al.*, [41]). Therefore, the computation of APCS for receptor modeling or source apportioning for each observation is required.

### APCS- MLR model

Basically MLR is based on a linear least-squares fitting process which requires a trace element or property to be determined for each source or source category in order to describe the old variables into new (Henry *et al.*, [42]). Therefore, the PCA and MLR were combined in order to identify the potential pollution sources of the Kuantan River Basin. Two basic types of receptor models that are generally applied for source apportionment are chemical mass balance (CMB) and multivariate techniques (Gordon, [43]). Other than that, PCA also identifies tracers that represent specific sources and the sources are selected as input (independent variables) to predict dependent variables (Morandi *et al.*, [44]). MLR are used particularly to explain the relationship between the source apportionment generated by PC and their correlation to WQI values. Other than that, MLR also examines the relationship of each source to the dependant variable (WQI) with five VFs as independent variables. The source apportionment is a vital environmetric technique as it estimates the contribution of identified sources to the concentrations of each parameter (Simeonov *et al.*, [35]). Sources of contributions were then calculated with APCS-MLR to identify main pollution origin in Kuantan River Basin after determining the number and characteristics of possible sources. The coefficient of determination (*R*^*2*^) is commonly used to evaluate model performance (Pearson, [45]); however *R*^*2*^ is not a good comparison measurement of different model since *R*^*2*^ only provides how excellent the model fits the data not how well it performs on external data (Aertsen *et al.*, [19]). Table 2 represents the MLR model and the goodness of fitting statistics.

**Table 2:** Summary of regression of variable WQI

**Goodness of fit statistics**	
Observations	275
Sum of weights	275
DF	269
R^2^	0.865
Adjusted R^2^	0.863
MSE	31.589
RMSE	5.62
AIC	955.454
SBC	977.155

**Figure 7:**
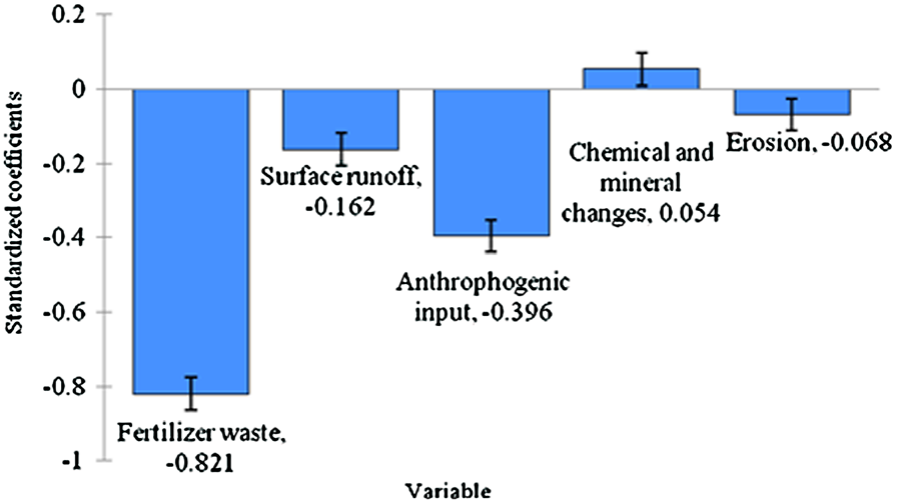
Standardized coefficients for each variable.

Figure 7 shows the standardized coefficients of independent variable of the WQI linear regression model and the contribution for each pollutant.

**Figure 8:**
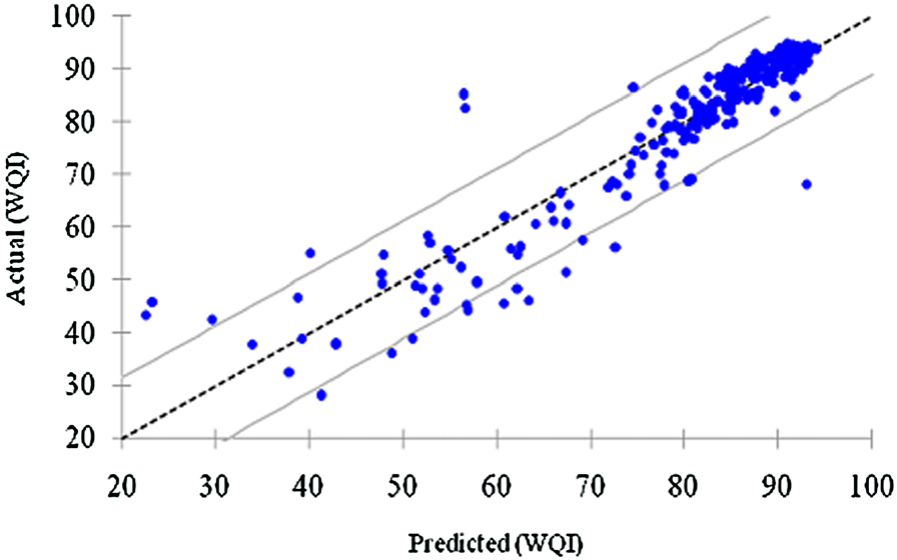
Distribution of predicted and actual WQI.

Figure 8 shows the graph for calculated WQI and predicted WQI. It is known that 19 observations from overall observations were out from the range of upper and lower boundary (95% mean of the confidence interval).

**Figure 9:**
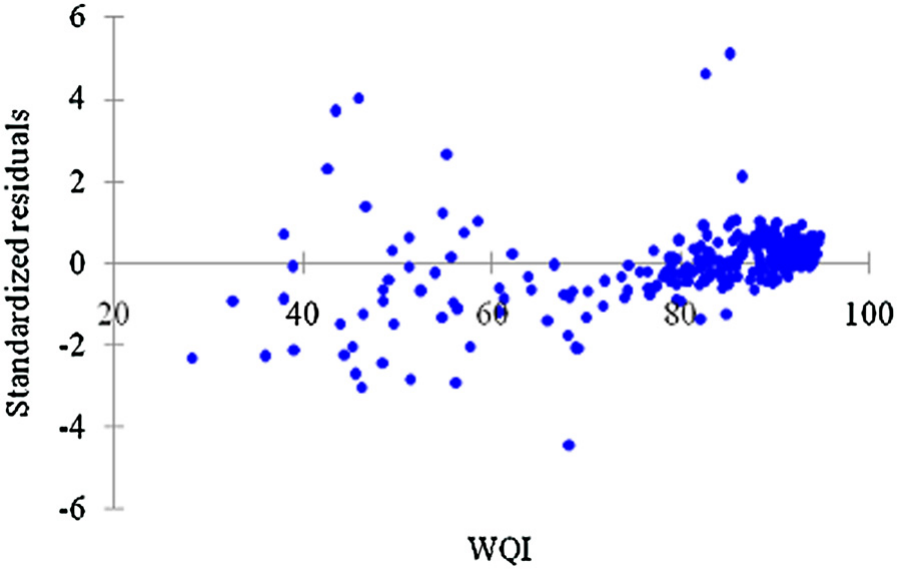
Residual between actual WQI and predicted WQI.

Figure 9 illustrates the residual analysis of the actual and predicted WQI using APCS-MLR model. The results show the deficiency of the APCS-MLR model as the data set with great difference in the range of −6 to 6.

### APCS-ANN

APCS-ANN is a comparatively new concept driven in river water quality modeling to allow non-linear relationships between variables to be ‘learnt’ through repeated presentation of input–output data sets. The use of numerical models such as ANN provides powerful tools to stimulate complex natural resources management problems (Nikolos *et al.*, [46]). Currently in environmental modeling, the aid of ANN to achieve good estimation and better accuracy in simulation and forecasting are beyond the typical model obtained when using entirely linear models. Other than that, ANN also allows one to resemble any mathematical function with absolute accuracy and used for non-linear regression between different variables in a self optimizing way. ANN has been conveniently applied in river water quality study at Langat River, Malaysia (Juahir *et al.*, [18]). Although PCA offered qualitative information about the major source of pollution to Kuantan River basin, it also provided the quantitative information on the pollutant contributor of each source types (Wu *et al.*, [47]).

**Figure 10:**
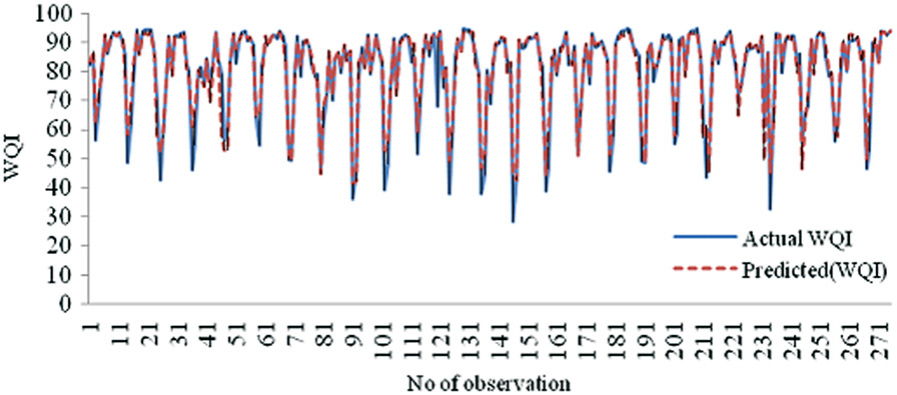
Estimation of predicted WQI and actual WQI.

Figure 10 demonstrates the performance of the ANN model of Kuantan River Basin representing the training and testing based on actual WQI and predicted WQI.

**Figure 11:**
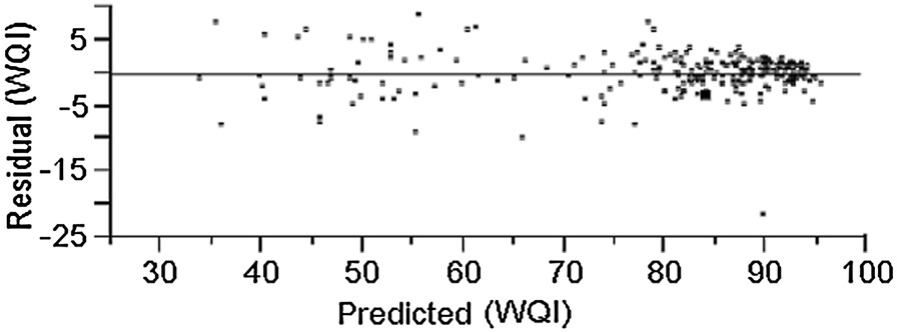
Residual graph.

Figure 11 represents the residual graph and shows the contrast of the actual and predicted WQI values. The residual values for each observation were in the range of −25 to 10.

### Determination of appropriate model APCS-ANN model based on sensitivity analysis

Classical process-based modeling approaches can provide good evaluations of water quality variables however the approach is too common to be applied directly without a lengthy data calibration process (Palani *et al.*, [48]). Since APCS-ANN gives better accuracy compared to APCS-MLR model, therefore detailed analysis is required for assessment in order to identify the effect of input variables towards the output. Sensitivity analysis was performed on the data set using varimax factor as the input and WQI values as the output layer. For the entire created network, four hidden layers were used as it been selected in optimal architecture of the input parameters. This is important to mention as ANN networks are sensitive to the number of hidden layer. Lesser number of hidden nodes may result under fitting in the model (Dogan *et al.*, [49]).

The results depicted in Table 3 show models performance evaluations of the effective parameters for forecasting WQI values. These models have been built through sensitivity analysis by removing one parameter or one model at a time. Sensitivity analysis is necessary to know how significant the excluded parameter or model would affect the *R*^*2*^ values (Lee *et al.*, [50]).

**Table 3:** The results of sensitivity analysis

**Model**	***R***^***2***^	**Difference *R***^***2***^	**Contribution (%)**	**RMSE**
All parameters	0.968			2.6409
L-FW	0.8115	0.1565	59.82	6.9275
L-SR	0.9329	0.0351	13.42	4.1094
L-AI	0.9092	0.0588	22.48	4.5495
L-CMC	0.9629	0.0051	1.95	2.8014
L-E	0.9619	0.0061	2.33	3.0198
Total		0.2616	100	

## Discussion

Based on Figure 5, PCA was applied to the data set to compare the compositional pattern between the analyzed water samples and to identify the factor that reflects with each other (Singh *et al.*, [1]). PCA was performed on the raw dataset comprising all the 13 water quality parameters (DO, BOD, COD, SS, pH, NH_3_-NL, DS, TS, NO_3_, Cl^-^, PO_4_, *E.coli*, coliform) with 275 observations to identify the pollution sources. PCA is able to describe the relationship between analytical variables than single analytical variable alone. VF1 (Eigenvalue 4.213) represents 21.40% of the total variability in one axis (VF1) comprising DO, AN and PO_4_. VF1 represents moderate loading matrix of coliform and *E. coli*. while DO was negatively correlated to AN and PO_4_ owing to the decrease of DO values in the increasing AN and PO_4_ inputs in the water body at Kuantan River. VF2 explain DS, TS and Cl in new variable with strong factor loadings.

According to Table 1 and Figure 6a, DO, AN and PO_4_ were strongly correlated to VF1 (32.409% of variance) and a new variable termed as fertilizer waste which explains that NH_4_ likely to comes from the vicinity of animal farm and agricultural nonpoint source (Crowther *et al.*, [51]; Singh *et al.*, [52]; Song *et al.*, [53]). Moderate loading of coliform and *E. coli* suggested minimum contribution of fecal pollution to the agriculture waste in Kuantan River Basin.

As shown in Table 1 and Figure 6b, surface runoff was named after VF2 (20.430% of variance) with high factor loadings for DS, TS and Cl^-^. While for VF3 (9.605% of variance) (Figure 6c) was strongly correlated with BOD and COD representing the influence of anthropogenic input typically organic pollution such as runoff from solids or waste disposal activities (Song *et al.*, [53]). VF4 (9.110% of variance) and VF5 (7.858% of variance) were completely different from the other VFs owing to only one parameter that significantly related to their corresponding axis (Figure 6d and e). Thus, VF4 and VF5 were named as chemical and mineral changes (pH) and erosion (SS), respectively. The new variables created were further introduced to two different numerical modeling networks for WQI prediction and apportioning the sources that contribute to Kuantan River Basin.

In this study, factor scores from PCA after varimax rotation were used in receptor models development using MLR and ANN. Both models were further compared to evaluate the performance on the data set. The use of PC based models was considered more dynamic, due to elimination of collinearity problems and prediction improvement (Sousa *et al.*, [8]). Moreover the utility of APCS that contain minimum input for both model compared to the raw data set was beneficial since it will increase the computational efficiency and interpretability and reduce the noise and redundancy for the model.

Referring to Table 2, the *R*^*2*^ value for APCS-MLR model in this study is 0.87 and the model indicates that 87% variability of WQI explained by the five independent variables used in the model. While for adjusted *R*^*2*^ it is always less than *R*^*2*^ and increases only if the new term improve the model (Aertsen *et al.*, [19]). Mean Square Error (MSE) and Root Mean Square Error (RMSE) measure residual errors which give estimation of the mean difference between observed and modeled values of WQI. The minimum value of MSE for APCS-MLR result (Table 2) corresponds to best network topology (Sousa *et al.*, [8]).

Best model performance are Akaike’s Information Criteria (AIC) and Schwarz Bayesian Criteria (SBC) values and *R*^*2*^ and adjusted *R*^*2*^ values closet to unity (Aertsen *et al.*, [19]). In general AIC, Bayesian Information Criteria (BIC) and SBC estimate the loss of accuracy caused by accounting a number of parameters and the number of data points used in its calibration. The small difference for AIC and SBC values signify that MLR was a fit method for WQI prediction. The high and great difference between values of AIC and SBC from the APCS-MLR model in this study (Table 2) indicate that the model has inadequacy in terms of fitness and robustness.

Based on Figure 7, fertilizer waste accounts as the highest pollution contributor to Kuantan River Basin while the next main contributor was anthropogenic input that may come from the vicinity area of Kuantan River basin. The negative standardized coefficient of independent variables (fertilizer waste, surface runoff, anthropogenic input and erosion) is based on negatively correlation to WQI values (as all the four independent variable decrease, WQI value increase). As shown in Figure 8, this proved that this model is able to predict WQI values from the varimax factor of PCA with negligible precision. In Figure 9, the verification and applicability of the model was influenced by the existence of the outlier observations as shown also in Figure 8.

APCS-ANN (WQI) was developed to investigate which pollution patterns contribute most to the Kuantan River Basin. Previously five VFs were generated from PCA after varimax rotation and the VFs were used as input parameter for ANN model. The five input parameters were fertilizer waste, surface runoff, and anthropogenic input, chemical and mineral changes and erosion and WQI as output. Based on Figure 10, APCS-ANN model developed produced good accuracy with *R*^*2*^ value, 0.9680 (Table 3- all input) for both training and testing sets with 66.76% and 33.33% of the overall data set. The correlation coefficients for both set approach to 1 which further explain the network output almost equal to the output (Garcia and Shigidi, [54]) and high accuracy for the cross validation with minimum value of RMSE (Rossel and Behrens, [14]). As shown in Figure 9 the predicted WQI values from the training set are able to follow the pattern recognized by the training set and produce high reliability and goodness-of-fit. The RMSE was chosen as main criteria to determine model performance. The APCS-ANN model has low value of RMSE (2.6409) compared to the APCS-MLR model (5.6200).

As shown in Figure 11, although the range is quite broad, the residual data were evenly distributed in the zero values. Only few outliers and extreme values were identified which only contribute minimum error to model robustness. As shown in Table 3, APCS-ANN model with all input parameters were selected as the most appropriate model for WQI forecasting with high *R*^*2*^ is (0.9680) and low RMSE (2.6409) as compared to other models. From the sensitivity analysis, the highest pollutant that contributed to Kuantan River Basin (WQI variation) was identified. Fertilizer waste (L-FW) accounted as the main pollution contributor (high percentage contribution, 59.82%) due to the exclusion of the parameters results in reduction of *R*^*2*^ (0.8115) and high RMSE (6.9275) which signify the model. Anthropogenic input (L-AI) was identified as the second pollution contributor (percentage contribution, 22.48%), *R*^*2*^ (0.9092) and RMSE (4.549) followed by surface runoff (L-SR), erosion (L-E) and lastly chemical and mineral changes (L-CMC) which were the least contributors as the inputs influencing the APCS-ANN model performance.

APCS-MLR in apportionment of sources affecting water quality reveals that industrial discharge contributed the highest pollutant of ammonia observed (Dalal *et al.*, [55]). However, application of in APCS-ANN Kuantan River Basin indicates a better accuracy than APCS-MLR shows that this is not an industrialized region yet it is governed by agriculture (palm oil plantation); thus fertilizer seems to be the major contributor. Therefore this study is expected to establish the baseline comparison in identifying the pollution contribution for future water resources and management.

## Conclusion

As a conclusion, agricultural practices and minimum contribution of anthropogenic human activities were among the responsible sources for surface water pollution in Kuantan River Basin, Malaysia. A main AN and PO_4_ inputs that reflects to DO reading from year 2003–2007, were due to the oil palm, rubber and forestry areas along the river. This also leads to chemical mineral changes to the field areas. From the results stated above, it is shown that ANN gives better accuracy as compared to MLR technique for WQI forecasting. Moreover ANN also capable to stimulate the complex relationship between the data set and consequently is able to justify the water quality puzzles. By using PCA, main pollution contributors to the basin were justified without eliminating any data and parameters. Moreover due to non-linearities of dependent variables in this study and the intricate associations between water quality parameters and WQI values, APCS-ANN model is able to justify and predict the WQI values at Kuantan River Basin. In this sense, APCS methods proved constitute recommended tools for more comprehensible of large volume data sets especially in environmental monitoring studies. Thus, the prediction of WQI values using APCS-ANN model can be used for environmental monitoring agencies in Malaysia to reduce the monitoring and chemical analysis cost as only significant parameters (DO, AN and PO_4_) will further used for monitoring purposes. The model gives efficient computational judgments.

## Competing interests

The authors declare that they have no competing interests.

## Authors’ contributions

Nasir MFM, carried out the environmental modeling studies, assisting in the writing, data analyzing, drafted and submission of the manuscript. Zali AM, carried out the environmental modeling studies, assisting in the writing, data analyzing and drafted the manuscript. Juahir H, carried out the environmental modeling studies, participated in sequence alignment and reviewing the manuscript. Hussain H, contributed in the data and landuse map of the manuscript. Zain SM, participated in the reviewing of the manuscript. Ramli N., contributed in the water quality data for the manuscript. All authors read and approved the final manuscript.
